# Supplementary material for: Clinical risk factors and blood protein biomarkers of 10-year pneumonia risk
Source: PLoS One. 2024 Jul 5;19(7):e0296139. doi: 10.1371/journal.pone.0296139 (PMC11226120; doi:10.1371/journal.pone.0296139)
Supplement: S1 File — (DOCX) [file pone.0296139.s001.docx]

**Clinical risk factors and blood protein biomarkers of 10-year pneumonia risk**

Ming-Ming Lee, MD; Yi Zuo, MPH; Katrina Steiling, MD, MSc; Joseph P. Mizgerd, ScD; Bindu Kalesan, PhD; Allan J. Walkey, MD, MSc

**Online Data Supplement**

**S1.** Calibration plots for the 4 risk models: A) a model including only clinical variables from the Framingham Heart Study clinical exam data; B) a model including only examination 7 serum protein immunoassay measurements; C) a model including both clinical variables and serum protein immunoassay measurements; D) an exploratory model including clinical covariates and routinely available C-reactive protein. The horizontal axis shows predicted risk of pneumonia, while the vertical axis shows the observed frequency of pneumonia. The 45-degree line represents perfect prediction. The predictions plotted about the 45-degree line in each model shows good calibration, except at extreme levels of pneumonia risk, with the exploratory model showing optimal calibration.

**
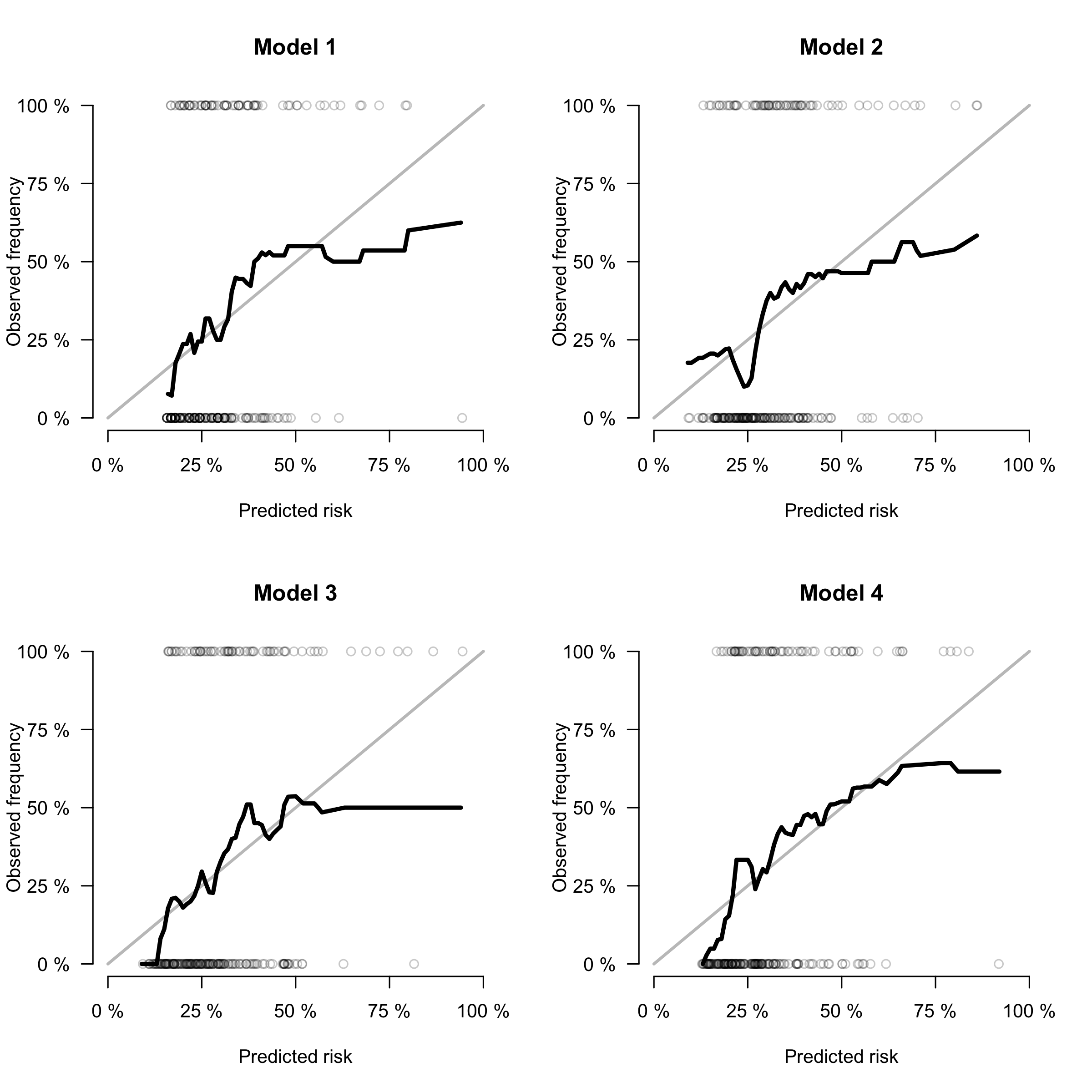
**

**S1 Table.** List of 88 protein immunoassays performed on blood drawn at examination 7.

| **Protein Immunoassay** |
| --- |
| Actin binding LIM protein 1* |
| Adipsin |
| Adrenomedullin |
| Alpha-1-acid glycoprotein 1 |
| Alpha-1-microglobulin |
| AMBP-bikunin |
| Aminolevulinate, delta-, synthase 2* |
| Angiopoietin-like 3 |
| Annexin A1* |
| Apolipoprotein A-1 |
| Apolipoprotein B |
| Apolipoprotein-A1 |
| Apolipoprotein-B100 |
| Beta-2-microglobulin |
| Butyrylcholine esterase |
| C-Reactive Protein |
| Cadherin-13 |
| CD5 antigen-like |
| Ceruloplasmin |
| Chemokine ligand 16 |
| Clusterin |
| Collagen, type XVIII, alpha 1 |
| Colony stimulating factor 2 receptor, beta, low-affinity (granulocyte-macrophage)* |
| Complement C2 |
| Contactin 1 |
| Cystatin-C |
| Dipeptidyl-pepridase 4 |
| Disintegrin and metalloproteinase domain-containing protein 15 |
| EGF containing fibulin-like extracellular matrix protein 1 |
| Enkurin* |
| Fibrinogen |
| Fibroblast growth factor 23 |
| FMS-related tyrosine kinase 3* |
| Glyceraldehyde 3-phosphate dehydrogenase |
| Glycoprotein V |
| Granule membrane protein 140 |
| Granulin |
| Granzyme B* |
| Growth differentiation factor 15 |
| Hemopexin |
| Iduronate 2-sulfatase* |
| Insulin-like growth factor 1 |
| Insulin-like growth factor 1 receptor* |
| Insulin-like growth factor binding protein 2 |
| Insulin-like growth factor-binding protein 1 |
| Insulin-like growth factor-binding protein 3 |
| Intercellular adhesion molecule 1 |
| Interleukin 6* |
| Interleukin-6 receptor beta |
| Leptin |
| Leptin receptor |
| Lipoprotein(a) |
| Lithostathine-1-alpha |
| Low density lipoprotein receptor |
| Matrix metallopeptidase 8 |
| Matrix metallopeptidase 9 |
| Melanoma cell adhesion molecule |
| Monocyte chemotactic molecule 1 |
| Monocyte differentiation antigen CD14 |
| Myelin P2 protein |
| Myeloperoxidase |
| Myoglobin |
| N-terminal prohormone of brain natriuretic peptide |
| Neural cell adhesion molecule |
| Neuronal cell adhesion molecule |
| Osteocalcin |
| Plasma kallikrein |
| Plasminogen activator inhibitor 1 |
| Pro-platelet basic protein |
| Protein Z-dependent protease inhibitor |
| Purine nucleoside phosphorylase* |
| Receptro for advanced glycation endproducts |
| Resistin |
| Runt-related transcription factor 1* |
| Scavenger receptor cysteine-rich type 1 protein |
| Serum amyloid A1 |
| Serum paraoxonase/arylesterase 1 |
| Soluble CD40 ligand |
| Stromal cell-derived factor 1 |
| Tetranectin |
| Thrombospondin-1* |
| Tissue inhibitor of metalloproteinases 1 |
| Translocation associated notch homolog |
| Troponin* |
| TSC22 domain family, member 3 |
| Uncarboxylated matrix gla protein |
| Vascular endothelial growth factor |
| WNK lysine deficient protein kinase 1 |

All proteins were measured from plasma in pg/mL. Luminex™ xMAP multiplex immunoassay technology was used to measure the biomarkers [1]. Samples were obtained between 7 to 9 a.m. after an overnight fast and stored at -80°C after centrifugation and until assay.

*Fourteen proteins were analyzed as binary variables defined as above or below a detection limit due to a high rate of missing data above the detection limit [2].

**S2 Table.** Univariate association between baseline clinical characteristics and pneumonia definitions using logistic regression model (N=1,370).

|  | **Definition 1***  **OR (95% CI)**  **(N=501)** | **Definition 2***  **OR (95% CI)**  **(N=218)** | **Definition 3***  **OR (95% CI)**  **(N=142)** | **Definition 4***  **OR (95% CI)**  **(N=400)** |
| --- | --- | --- | --- | --- |
|  |  |  |  |  |
| **Demographics** |  |  |  |  |
| Age | 1.08 (1.05, 1.10) | 1.06 (1.03, 1.10) | 1.05 (1.02, 1.09) | 1.07 (1.05, 1.10) |
| Female | 0.91 (0.73, 1.14) | 0.79 (0.59, 1.06) | 0.87 (0.61, 1.22) | 0.88 (0.70, 1.12) |
| Marital status | 1.03 (0.81, 1.31) | 1.14 (0.83, 1.58) | 1.07 (0.73, 1.57) | 0.90 (0.70, 1.16) |
| Current smoker | 1.67 (1.14, 2.45) | 2.01 (1.29, 3.14) | 1.57 (0.91, 2.71) | 1.84 (1.25, 2.72) |
| Alcohol use | 0.97 (0.66, 1.43) | 0.82 (0.48, 1.40) | 0.65 (0.32, 1.31) | 0.86 (0.57, 1.30) |
|  |  |  |  |  |
| **Clinical comorbidities** |  |  |  |  |
| Atrial fibrillation/flutter | 1.98 (1.42, 2.77) | 2.01 (1.36, 2.97) | 1.83 (1.15, 2.90) | 1.73 (1.23, 2.44) |
| Cancer | 1.10 (0.83, 1.45) | 1.03 (0.71, 1.49) | 1.15 (0.75, 1.77) | 1.00 (0.74, 1.35) |
| Chronic kidney disease | 1.54 (1.18, 2.00) | 1.68 (1.21, 2.34) | 1.66 (1.13, 2.45) | 1.44 (1.09, 1.91) |
| Chronic pulmonary disease | 2.57 (1.65, 4.01) | 3.72 (2.34, 5.90) | 4.95 (3.03, 8.08) | 2.88 (1.85, 4.74) |
| Depression | 1.26 (0.99, 1.62) | 1.26 (0.91, 1.73) | 1.15 (0.78, 1.69) | 1.43 (1.11, 1.85) |
| Diabetes | 1.50 (1.12, 2.02) | 1.51 (1.04, 2.19) | 1.27 (0.80, 2.02) | 1.25 (0.92, 1.71) |
| Heart failure | 2.71 (1.56, 4.71) | 2.71 (1.51, 4.86) | 2.26 (1.14, 4.48) | 2.63 (1.53, 4.52) |
| Hip fracture | 1.88 (0.92, 3.83) | 1.87 (0.83, 4.24) | 2.12 (0.86, 5.27) | 2.32 (1.14, 4.75) |
| Hypertension | 1.37 (1.09, 1.73) | 1.69 (1.22, 2.33) | 1.57 (1.06, 2.31) | 1.21 (0.94, 1.54) |
| Peripheral vascular disease | 1.39 (0.85, 2.29) | 1.83 (1.02, 3.29) | 1.85 (0.94, 3.64) | 1.74 (1.05, 2.88) |
| Pulmonary embolus | 6.97 (0.78, 62.7) | 3.55 (0.59, 21.3) | - | 3.66 (0.61, 22.0) |
| Deep vein thrombosis | 1.74 (0.61, 5.00) | 0.40 (0.01, 3.10) | 2.39 (0.66, 8.66) | 0.97 (0.30, 3.11) |
| Myocardial infarction | 1.06 (0.73, 1.54) | 1.49 (0.95, 2.32) | 1.03 (0.57, 1.84) | 1.10 (0.75, 1.63) |
| Valve disease | 1.25 (0.94, 1.67) | 1.63 (1.14, 2.33) | 1.44 (0.93, 2.22) | 1.21 (0.89, 1.64) |
|  | |  |  |  |
| **Cognitive and functional status** | |  |  |  |
| Mini-mental state exam | 0.99 (0.96, 1.01) | 1.00 (0.96, 1.04) | 0.99 (0.95, 1.03) | 0.98 (0.95, 1.01) |
| Kannel physical activity index | 0.99 (0.97, 1.01) | 0.99 (0.96, 1.01) | 0.98 (0.95, 1.01) | 0.99 (0.97, 1.01) |
| Activities of daily living score | 1.03 (0.89, 1.20) | 1.03 (0.84, 1.27) | 0.92 (0.75, 1.13) | 1.01 (0.87, 1.19) |
| Residence place |  |  |  |  |
| Other institution | Reference | Reference | Reference | Reference |
| Private residence | 0.88 (0.28, 2.69) | 0.35 (0.04, 2.97) | 1.59 (0.27, 9.53) | 0.86 (0.26, 2.80) |
| Nursing home | 1.00 (0.57, 1.76) | 1.12 (0.52, 2.41) | 1.49 (0.53, 4.18) | 0.91 (0.51, 1.64) |
| Physical disability | 1.73 (1.13, 2.66) | 1.92 (1.17, 3.16) | 3.14 (1.88, 5.27) | 1.96 (1.27, 3.03) |
| Overnight hospitalization in the past year |  |  |  |  |
| No | Reference | Reference | Reference | Reference |
| Yes, once | 1.58 (1.20, 2.06) | 1.30 (0.91, 1.84) | 1.34 (0.88, 2.04) | 1.62 (1.22, 2.14) |
| Yes, more than once | 1.64 (1.14, 2.37) | 1.61 (1.03, 2.52) | 2.05 (1.25, 3.39) | 1.60 (1.10, 2.34) |
|  |  |  |  |  |
| **Other measures** |  |  |  |  |
| Heart rate | 1.00 (0.99, 1.01) | 1.01 (0.99, 1.02) | 1.01 (1.00, 1.03) | 1.00 (0.99, 1.01) |
| Mean arterial pressure | 1.00 (0.99, 1.00) | 1.00 (0.99, 1.01) | 1.00 (1.00, 1.01) | 1.00 (0.99, 1.00) |
| Body mass index category | 1.03 (0.90, 1.19) | 0.96 (0.79, 1.16) | 1.05 (0.83, 1.31) | 0.97 (0.83, 1.13) |

*Definition of abbreviations*: CI = confidence interval; OR = odds ratio.

*Definition 1 = any pneumonia (ICD-9-CM codes for viral, pneumococcal, other bacterial, bronchopneumonia – organism unspecified, pneumonia – organism unspecified, and influenza) in any clinical setting; definition 2 = any pneumonia in the inpatient setting; definition 3 = bacterial pneumonia in any clinical setting; definition 4 = non-viral pneumonia in any clinical setting. Definition 2 refers to community-acquired pneumonia requiring hospitalization and not hospital-acquired pneumonia.

**S3. Table** Definitions of Framingham Heart Study covariates.

|  | **Data Source** | **Definition** |
| --- | --- | --- |
| **Demographics** |  |  |
| Age | Exam | Years |
| Sex | Exam | Male or female |
| Marital status | Exam | Single, married, widowed, divorced or separated |
| Residence place | Exam | Private residence, nursing home, other institution (such as home-self care, retirement village) or unknown |
| Current smoker | Exam | Smoked cigarettes regularly within the year prior to exam |
| Alcohol use | Exam | ≥14 drinks per week (men), ≥ 7 drinks per week (women) |
|  |  |  |
| **Clinical comorbidities** |  |  |
| Atrial fibrillation/flutter | Sequence of events | Present on electrocardiogram from FHS clinical exam, external physician visit, hospital records or Holter report |
| Cancer (excluding non-melanomatous skin cancer) | Sequence of events | Documentation of cancer from records from health care providers and from other registries, coded by location of primary cancer and malignant |
| Chronic kidney disease | Exam | Creatinine >1.3 (men) and Creatinine >1.0 (women) = CrCl <60 |
| Chronic pulmonary disease | Exam | Clinical diagnostic impression of emphysema or chronic bronchitis |
| Diabetes | Exam | Fasting glucose ≥ 126 mg/dl, random glucose ≥ 200 mg/dl or participant prescribed medication for diabetes |
| Depression | Exam | Participant prescribed medication for depression or CES-D score ≥ 16 |
| Heart failure | Sequence of Events | Diagnosed on basis of exam or physician notes. Diagnosis of congestive heart failure (CHF) required a minimum of 2 major or 1 major and 2 minor criteria present concurrently. The presence of other conditions capable of producing the symptoms and signs were considered in evaluating the findings. Major criteria: 1) paroxysmal nocturnal dyspnea or orthopnea; 2) distended neck veins (in other than the supine position); 3) rales; 4) increasing heart size by x-ray; 5) acute pulmonary edema on chest x-ray; 6) ventricular S3 gallop; 7) increased venous pressure >16 cm H_2_O; 8) hepatojugular reflux; 9) pulmonary edema, visceral congestion, cardiomegaly shown on autopsy; weight loss on CHF medication of 10 lbs over 5 days. Minor criteria: 1) bilateral ankle edema; 2) night cough; 3) dyspnea on ordinary exertion; 4) hepatomegaly; 5) pleural effusion by x-ray; 6) decrease in vital capacity by one-third from maximum record; 7) tachycardia (120 bpm or more); 8) pulmonary vascular engorgement on chest x-ray. |
| Hip fracture | Sequence of Events | History of lifetime hip fracture(s) |
| Hypertension | Exam | SBP ≥ 140 mmHg, DBP ≥ 90 mmHg or participant prescribed medication for hypertension |
| Peripheral vascular disease | Sequence of Events | Subjective intermittent claudication defined by a cramping discomfort in the calf clearly provoked by walking some distance with pain appearing sooner when walking quickly or uphill and relieved within a few minutes by rest. Diagnosis was made by 2 physicians at a Framingham clinic visit or the Framingham Endpoint Review Committee. |
| Pulmonary embolus/deep vein thrombosis | Sequence of Events | PE by pulmonary arteriogram, CTPA, MRI; PE by history and high probability V/Q scan; PE by clinical high suspicion and documented source of PE; DVT by venogram, doppler ultrasound, impedance plethysmography, fibrinogen leg scan or CT. Definite PE/DVT was coded if the Framingham Endpoint Committee agreed that PE or DVT was present upon review of medical records. |
| Myocardial infarction | Sequence of Events | MI recognized with diagnostic ECG, MI recognized without diagnostic ECG but with enzymes and history, MI recognized without diagnostic ECG but with autopsy evidence. Recognized means MI was known to have occurred at time of MI occurrence. |
| Valvular disease | Exam | Presence of ≥3 of 6 systolic murmur or any diastolic murmur on exam |
| Stroke | Sequence of Events | Diagnosis of cerebrovascular disease is based on the occurrence of a clinically evidence stroke documented by clinical records reviewed by at least 2 neurologists. Stroke is defined as the sudden or rapid onset of a focal neurologic deficit persisting for >24 hours. Stroke is further categorized into hemorrhagic stroke (e.g., subarachnoid hemorrhage, intracerebral hemorrhage), ischemic stroke (e.g., cerebral embolism), atherothrombotic brain infarction, or transient ischemic attack. |
|  |  |  |
| **Cognitive and Functional Status** | |  |
| Mini-mental state exam | Exam | Mini-mental state examination |
| Kannel physical activity index | Exam | Kannel physical activity index questionnaire |
| Activities of daily living score | Exam | Katz index of independence in activities of daily living questionnaire |
| Physical disability | Exam | Human assistance needed/minimally dependent, dependent or not performed during a normal day in response to walking about 50 yards on level surface or walking up and down one flight of stairs |
| Overnight hospitalization in past year | Exam | Yes-once, yes-more than once, or no |
|  |  |  |
| **Other measures** |  |  |
| Heart rate | Exam | Beats per minute |
| Systolic blood pressure | Exam | mmHg |
| Diastolic blood pressure | Exam | mmHg |
| Body Mass Index | Exam | Height/weight^2^  <18.5 underweight  18.5-24.9 normal  25-29.9 overweight  30-39.9 obese  ≥40, morbid obesity |

*Definition of abbreviations:* CrCl = creatinine clearance, CES-D = center for epidemiologic studies-depression, CT = computed tomography, CTPA = computed tomography pulmonary angiogram, DBP = diastolic blood pressure, DVT = deep vein thrombosis; MI = myocardial infarction; MRI = magnetic resonance imaging; PE = pulmonary embolus; SBP = systolic blood pressure, V/Q scan = ventilation/perfusion scan.

# S4. Table. Sensitivity analyses showing multivariate association between baseline clinical covariates and pneumonia using Cox proportional hazard models with four different approaches to variable selection (N=1,370).

|  | **Model 1***  **HR (95% CI)** | **Model 2***  **HR (95% CI)** | **Model 3***  **HR (95% CI)** | **Model 4***  **HR (95% CI)** |
| --- | --- | --- | --- | --- |
| **C-statistic** | 0.69 | 0.67 | 0.69 | 0.67 |
| **Akaike information criterion** | 5069.72 | 5918.01 | 5053.08 | 5906.00 |
|  |  |  |  |  |
| **Demographics** |  |  |  |  |
| Age | 1.10 (1.07, 1.12) | 1.10 (1.07, 1.12) | 1.10 (1.07, 1.12) | 1.09 (1.07, 1.11) |
| Sex |  |  |  |  |
| Male | Reference |  |  |  |
| Female | 0.90 (0.72, 1.13) |  |  |  |
| Marital status | 1.40 (1.09, 1.80) | 1.35 (1.08, 1.67) | 1.40 (1.10, 1.78) | 1.35 (1.09, 1.68) |
| Residence place |  |  |  |  |
| Other institution | Reference |  |  |  |
| Private residence | - |  |  |  |
| Nursing home | 1.22 (0.71, 2.08) |  |  |  |
| Current smoker | 1.77 (1.20, 2.60) | 1.73 (1.23, 2.45) | 1.71 (1.17, 2.51) | 1.75 (1.24, 2.46) |
| Alcohol use | 1.43 (1.04, 1.98) | 1.22 (0.89, 1.66) | 1.40 (1.02, 1.93) | 1.22 (0.89, 1.66) |
|  |  |  |  |  |
| **Clinical comorbidities** |  |  |  |  |
| Atrial fibrillation/flutter | 1.73 (1.25, 2.38) | 1.57 (1.19, 2.09) | 1.77 (1.30, 2.40) | 1.57 (1.18, 2.08) |
| Cancer | 1.21 (0.94, 1.57) |  | 1.26 (0.98, 1.62) |  |
| Chronic kidney disease | 1.26 (0.94, 1.69) |  | 1.27 (0.96, 1.69) |  |
| Chronic pulmonary disease | 2.08 (1.43, 3.00) | 2.09 (1.48, 2.93) | 2.16 (1.50, 3.10) | 2.12 (1.51, 2.98) |
| Diabetes | 1.39 (1.07, 1.81) | 1.42 (1.13, 1.79) | 1.42 (1.10, 1.83) | 1.35 (1.07, 1.71) |
| Heart failure | 1.69 (1.00, 2.84) | 2.07 (1.34, 3.21) | 1.69 (1.04, 2.75) | 2.04 (1.32, 3.13) |
| Hip fracture | 1.18 (0.62, 2.27) |  |  |  |
| Hypertension | 1.29 (0.99, 1.66) |  | 1.33 (1.03, 1.71) |  |
| Peripheral vascular disease | 1.11 (0.73, 1.68) |  |  |  |
| Depression | 1.08 (0.86, 1.37) |  |  |  |
| Pulmonary embolus | 1.57 (0.37, 6.63) |  |  |  |
| Deep vein thrombosis | 1.13 (0.43, 2.97) |  |  |  |
| Myocardial infarction | 0.80 (0.55, 1.17) |  |  |  |
| Valvular disease | 1.17 (0.91, 1.50) |  |  |  |
|  |  |  |  |  |
| **Cognitive and functional status** | |  |  |  |
| Mini-mental state exam | 0.98 (0.94, 1.04) |  |  |  |
| Kannel physical activity index | 1.00 (0.98, 1.01) |  |  |  |
| Activities of daily living score | 0.52 (0.24, 1.14) |  | 0.45 (0.21, 0.96) | 0.92 (0.78, 1.08) |
| Physical disability | 1.30 (0.70, 2.39) |  |  |  |
| Overnight hospitalization in the past year |  |  | 1.23 (1.05, 1.43) | 1.22 (1.06, 1.41) |
| No | Reference | Reference |  |  |
| Yes, once | 1.50 (1.18, 1.90) | 1.42 (1.14, 1.77) |  |  |
| Yes, more than once | 1.23 (0.84, 1.81) | 1.34 (0.96, 1.86) |  |  |
|  |  |  |  |  |
| **Other measures** |  |  |  |  |
| Heart rate | 1.01 (1.00, 1.02) |  | 1.01 (1.00, 1.02) |  |
| Mean arterial pressure | 1.00 (0.99, 1.00) |  | 1.00 (0.99, 1.00) |  |
| Body mass index category | 1.02 (0.90, 1.16) |  |  |  |

*Definition of abbreviations:* CI = confidence interval; HR = hazard ratio.

*Model 1 includes all clinical covariates; model 2 includes only significant covariates from model 1; model 3 includes covariates with *p*-value <0.20 from model 1; model 4 includes only significant covariates from model 3.

# S5 Table. Multivariate association between baseline clinical covariates and pneumonia using competing risk survival model (N = 1,370).

|  | **cMVfull**  **SHR (95% CI)** | **cMVfull_1**  **SHR (95% CI)** | **cMV1**  **SHR (95% CI)** | **cMV2**  **SHR (95% CI)** |
| --- | --- | --- | --- | --- |
| **Akaike information criterion** | 5180.04 | 6052.70 | 5158.21 | 6041.23 |
|  |  |  |  |  |
| **Demographics** |  |  |  |  |
| Age | 1.09  (1.06, 1.12) | 1.08  (1.06, 1.10) | 1.09  (1.06, 1.11) | 1.08  (1.05, 1.10) |
| Sex |  |  |  |  |
| Male | Reference |  |  |  |
| Female | 0.96  (0.76, 1.21) |  |  |  |
| Marital status | 1.40  (1.08, 1.80) | 1.29  (1.03, 1.61) | 1.38  (1.09, 1.76) | 1.29  (1.03, 1.61) |
| Residence place |  |  |  |  |
| Other institution | Reference |  |  |  |
| Private residence | - |  |  |  |
| Nursing home | 1.07  (0.65, 1.78) |  |  |  |
| Current smoker | 1.73  (1.17, 2.58) | 1.56  (1.09, 2.22) | 1.73  (1.17, 2.56) | 1.55  (1.09, 2.22) |
| Alcohol use | 1.41  (1.01, 1.96) | 1.22  (0.89, 1.67) | 1.42  (1.02, 1.97) | 1.22  (0.89, 1.67) |
|  |  |  |  |  |
| **Clinical comorbidities** |  |  |  |  |
| Atrial fibrillation/flutter | 1.49  (1.04, 2.13) | 1.36  (1.01, 1.84) | 1.54  (1.10, 2.17) | 1.36  (1.01, 1.84) |
| Cancer | 1.10  (0.84, 1.44) |  |  |  |
| Chronic kidney disease | 1.21  (0.89, 1.66) |  | 1.23  (0.92, 1.65) |  |
| Chronic pulmonary disease | 1.74  (1.20, 2.53) | 1.83  (1.29, 2.60) | 1.71  (1.16, 2.52) | 1.83  (1.28, 2.61) |
| Diabetes | 1.33  (1.02, 1.75) | 1.30  (1.02, 1.66) | 1.36  (1.05, 1.78) | 1.25  (0.98, 1.60) |
| Heart failure | 1.16  (0.58, 2.30) |  |  |  |
| Hip fracture | 1.34  (0.68, 2.64) |  |  |  |
| Hypertension | 1.27  (0.97, 1.66) |  | 1.30  (1.00, 1.70) | 1.16  (0.94, 1.44) |
| Peripheral vascular disease | 1.12  (0.71, 1.77) |  |  |  |
| Depression | 1.05  (0.83, 1.34) |  |  |  |
| PE | 2.46  (0.48, 12.6) |  |  |  |
| DVT | 0.84  (0.25, 2.77) |  |  |  |
| MI | 0.73  (0.49, 1.10) |  | 0.77  (0.53, 1.12) |  |
| Valve disease | 1.07  (0.83, 1.40) |  |  |  |
|  |  |  |  |  |
| **Cognitive and functional status** | |  |  |  |
| Mini-mental state exam | 1.00  (0.95, 1.07) |  |  |  |
| Kannel physical activity index | 1.00  (0.99, 1.02) |  |  |  |
| Activities of daily living score | 0.82  (0.45, 1.51) |  |  |  |
| Physical disability | 1.12  (0.56, 2.24) |  |  |  |
| Overnight hospitalization in the past year |  |  | 1.20  (1.03, 1.41) | 1.17  (1.01, 1.35) |
| No | Reference | Reference |  |  |
| Yes, once | 1.47  (1.16, 1.87) | 1.38  (1.11, 1.73) |  |  |
| Yes, more than once | 1.14  (0.76, 1.72) | 1.20  (0.85, 1.71) |  |  |
|  |  |  |  |  |
| **Other measures** |  |  |  |  |
| Heart rate | 1.01  (1.00, 1.02) |  | 1.01  (1.00, 1.01) |  |
| Mean arterial pressure | 1.00  (0.99, 1.00) |  | 1.00  (0.99, 1.00) |  |
| Body mass index category | 1.05  (0.92, 1.20) |  |  |  |

*Definition of abbreviations:* CI = confidence interval; SHR = subdistribution hazard ratio.

*Model 1 includes all clinical covariates; model 2 includes only significant covariates from model 1; model 3 includes covariates with *p*-value <0.20 from model 1; model 4 includes only significant covariates from model 3.

**S6. Table.** Comparison of the pooled estimates after imputation and the estimates using complete cases of the association between baseline clinical covariates and pneumonia using competing risk model with LASSO variable selection (N=1,370).

| **Pooled estimates** | | | | |  | **Estimates using complete cases**  **(N = 1,182)** | | | | |
| --- | --- | --- | --- | --- | --- | --- | --- | --- | --- | --- |
| **Covariates** |  | **SHR** | **95% CI** | ***p*-value** |  | **Covariates** |  | **SHR** | **95% CI** | ***p*-value** |
| Age |  | 1.06 | (1.04, 1.08) | <0.0001 |  | Age |  | 1.09 | (1.06, 1.12) | <0.0001 |
| AF |  | 1.44 | (1.06, 1.95) | 0.018 |  | AF |  | 1.66 | (1.19, 2.31) | 0.003 |
| Chronic kidney disease |  | 1.14 | (0.89, 1.47) | 0.30 |  | Chronic kidney disease |  | 1.29 | (0.95, 1.74) | 0.10 |
| Chronic pulmonary disease |  | 1.89 | (1.34, 2.66) | 0.0003 |  | Chronic pulmonary disease |  | 1.78 | (1.20, 2.65) | 0.004 |
| Diabetes |  | 1.35 | (1.05, 1.73) | 0.020 |  | Diabetes |  | 1.48 | (1.13, 1.95) | 0.005 |
| Heart failure |  | 1.68 | (1.05, 2.67) | 0.029 |  | Marital status |  | 1.47 | (1.12, 1.94) | 0.005 |
| Hospitalization (Yes vs. No) |  | 1.29 | (1.05, 1.59) | 0.018 |  | Hospitalization (Yes vs. No) |  | 1.39 | (1.10, 1.77) | 0.007 |
| Smoking |  | 1.74 | (1.26, 2.39) | 0.0007 |  | Smoking |  | 2.03 | (1.36, 3.04) | 0.0001 |
| Physical disability |  | 1.16 | (0.79, 1.68) | 0.46 |  |  |  |  |  |  |

*Definition of abbreviations:* AF = atrial fibrillation/flutter; CI = confidence interval; SHR = subdistribution hazard ratio

**S7 Table.** **Clinical factors and serum protein biomarkers associated with pneumonia in the inpatient setting using the Fine-Gray competing risk model with multiple imputation and least absolute shrinkage and selection operator variable selection (N=218). We used the same risk models used for the primary analyses: A) a model including only clinical variables from the Framingham Heart Study clinical exam data; B) a model including only examination 7 serum protein immunoassay measurements; C) a model including both clinical variables and serum protein immunoassay measurements; and D) an exploratory model including clinical covariates and C-reactive protein.**

|  | A | | B | | | C | | | D | | |
| --- | --- | --- | --- | --- | --- | --- | --- | --- | --- | --- | --- |
|  | SHR | 95% CI | | SHR | 95% CI | | SHR | 95% CI | | SHR | 95% CI |
| Clinical covariates |  |  | |  |  | |  |  | |  |  |
| Age | 1.07 | (1.04, 1.09) | |  |  | | 1.05 | (1.03, 1.08) | | 1.07 | (1.04, 1.10) |
| Atrial fibrillation/flutter | 1.50 | (1.01, 4.30) | |  |  | | 1.62 | (1.11, 2.38) | | 1.44 | (0.96, 2.17) |
| Chronic pulmonary disease | 2.88 | (1.93, 4.30) | |  |  | | 3.02 | (2.03, 4.50) | | 2.73 | (1.83, 4.06) |
| Current smoking | 1.87 | (1.23, 2.82) | |  |  | |  |  | | 1.76 | (1.16, 2.66) |
| Diabetes | 1.43 | (1.01, 2.01) | |  |  | |  |  | | 1.39 | (0.99, 1.94) |
| Heart failure | 1.91 | (1.07, 3.42) | |  |  | |  |  | | 1.95 | (1.09, 3.48) |
| Hospitalization within 1 year | 1.13 | (0.94, 1.37) | |  |  | |  |  | | 1.11 | (0.92, 1.35) |
|  |  |  | |  |  | |  |  | |  |  |
| Immunoassay covariates |  |  | |  |  | |  |  | |  |  |
| log (C-reactive protein) |  |  | | 1.23 | (1.09, 1.39) | | 1.21 | (1.07, 1.36) | | 1.26 | (1.12, 1.42) |
| log (growth differentiation factor 15) |  |  | | 1.79 | (1.31, 2.45) | | 1.64 | (1.18, 2.28) | |  |  |
| log (matrix metallopeptidase 8) |  |  | | 1.26 | (1.07, 1.48) | | 1.26 | (1.06, 1.50) | |  |  |
| log(insulin-like growth factor binding protein 2) |  |  | | 1.22 | (0.92, 1.62) | |  |  | |  |  |
| log (N-terminal prohormone of brain natriuretic peptide) |  |  | | 1.08 | (0.94, 1.24) | |  |  | |  |  |
|  |  |  | |  |  | |  |  | |  |  |
| Akaike information criterion | 2502 | | 2531 | | | 2490 | | | 2495 | | |
| C-statistic | 0.67 | | 0.64 | | | 0.69 | | | 0.68 | | |

*Definition of abbreviations:* CI = confidence interval; SHR = subdistribution hazard ratio

**REFERENCES**

1. Johannisson A, Jonasson R, Dernfalk J, Jensen-Waern M. Simultaneous detection of porcine proinflammatory cytokines using multiplex flow cytometry by the xMAP technology. *Cytometry A.* 2006;69A:391–5.

2. Ho JE, Lyass A, Courchesne P, et al. Protein Biomarkers of Cardiovascular Disease and Mortality in the Community. *J Am Heart Assoc.* 2018;7:e008108.
