# Supplementary material for: Clinical risk factors and blood protein biomarkers of 10-year pneumonia risk
Source: PLoS One. 2024 Jul 5;19(7):e0296139. doi: 10.1371/journal.pone.0296139 (PMC11226120; doi:10.1371/journal.pone.0296139)
Supplement: Attachment — Submitted filename: PLOS ONE FHS CMS response to reviewers.docx [file pone.0296139.s002.docx]

Dear Editor,

Thank you very much for the reviews of our manuscript. We have revised the manuscript in response to the reviewer comments. Please see our point-by-point responses below detailing the revisions made in response to the reviewer comments.

Regards,

Allan Walkey, for the authors

Reviewer #1: Congratulations to the authors for their study. The study has novel feature that highlights the inflammatory markers predict the future pneumonia. The results obtained are well discussed in discussion section. In general, the manuscript has well written. particularly important bias points are properly addressed in the limitation. However, I will have some correction suggestions, follow as;
Query 1-"we did not observe chronic renal disease, cancer, BMI, alcohol use and nursing home residence to be independently predictive of future pneumonia risk potentially related to adjustment for more covariates and use of competing risk models in our study. Despite studies showing an association between cognitive impairment and pneumonia we interestingly did not find mini-mental status scores to be predictive of future pneumonia." On what do they base their results that contradict previous studies? An explanation can be added.

Response 1. Thank you for your review and for asking for clarification. We have added the following sentence to the Discussion to potentially explain some differences between our results and prior studies.

Manuscript change:

“The adjustment for additional clinical covariates and potential confounders in our study as compared with prior studies, which may be partially collinear with some comorbidities, as well as the use of competing risk statistical models, may explain some of the differences between our results and prior studies of pneumonia risk factors.”

Q2-On the Table 1, comorbidities should be arranged in order of incidence.

R2. Thank you, we have now listed the comorbidities in descending order.

Q3-Since the resolutions of the figures in the file are low, the texts cannot be clearly seem.

R3. Thank you we have increased the size of the fonts in all figures.
